# Refugee mental health and the role of place in the Global North countries: A scoping review

**DOI:** 10.1016/j.healthplace.2023.102964

**Published:** 2023-01

**Authors:** Guntars Ermansons, Hanna Kienzler, Zara Asif, Peter Schofield

**Affiliations:** a Department of Global Health & Social Medicine, School of Global Affairs, King's College London, 40 Aldwych, Bush House (NE), London, WC2B 4BG, UK; b Department of Population Health Sciences, School of Life Course & Population Sciences, King's College London, 3rd Floor, Addison House, Guy's Campus, London, SE1 1UL, UK

**Keywords:** Refugees, Mental health, Place, Post-migration, Scoping review

## Abstract

Post-migration factors significantly influence refugee mental health. This scoping review looks at the role of place in refugee mental health. We included 34 studies in Global North high-income countries that elaborated on the place characteristics of facilities, neighbourhoods, urban and rural areas, and countries. While the role of place remains under-theorised, all studies reveal common characteristics that support a strong relationship between place of residence, refugee mental health and wellbeing outcomes in post-migration context. Given that refugees often have little or no choice of where they ultimately live, we suggest future research should focus on how characteristics of place co-constitute post-migration refugee mental health risks, protections, and outcomes.

## Highlights

• Very few studies conceptualize place explicitly in relation to refugee mental health.
• Five place-specific key themes have been identified in the current research.
• Intersecting material, social and policy characteristics of place are relevant for refugee mental health.
• Place must play more central role in research and understanding of refugee mental health.

## 1 Introduction

This scoping review assesses current literature regarding the main characteristics of place and the pathways linking place to refugee mental health in post-migration contexts, with a particular focus on the Global North.^1^ Of the 82.4 million people currently displaced, 73% are in neighbouring, low- and middle-income countries (LMICs). Only a relatively small number of refugees are hosted by high-income countries, mostly in the Global North (UNHCR, 2020). Global North countries provide unique conditions grounded in economic, cultural, and socio-political realities that differ from those countries where most refugees are located, many in refugee camps. While arriving in a relatively wealthy host country and receiving refugee status may be a relief, it is often accompanied by difficulties relating to the places where refugees reside and try to settle (Darling, 2017; Nolas et al., 2020; Spicer, 2008).

Local policies affecting where refugees can live, and how they can interact with their new environments and host societies differ between and within countries. For instance, after gaining asylum in the UK, refugees have only 28 days until their asylum support expires, during which time they must find employment and private accommodation or apply for welfare and housing support (Citizens Advice, 2020). Such a rapid shift from severely limited social and economic options to full responsibility for one's own livelihood has been shown to be highly stressful (Rowley et al., 2020). By comparison, in Denmark, state and local authorities provide hands-on support and refugees are subject to mandatory integration programmes (Rytter, 2018). Thus, refugee vulnerability post-migration and their longer-term residence is a uniquely modifiable exposure – subject to the effects of asylum seeker dispersal policies, integration programmes and onward migration, particularly in the Global North (Stewart and Shaffer, 2015; van Liempt, 2011). It is therefore important to examine the differing place-related factors relevant to where refugees live, how structural conditions translate into everyday contexts, and their significance for refugee mental health and wellbeing.

The 1951 Refugee Convention considers refugees to be “persons who have escaped their country due to war, violence, persecution or natural disaster.” They are unable to return to their home countries because it is too dangerous, so they require to be hosted elsewhere (UNHCR, 2020). This review is focusing specifically on people who have received legal recognition of refugee status in Global North countries. That is, we do not focus on asylum seekers, internally displaced or stateless persons because their links to and experience of places are often significantly different. By ‘post-migration context’ we refer to the period after claims to asylum have been granted in a host country. This period may still involve international or domestic onward migration (Shaffer and Stewart, 2021). ‘Place’ we define as locations where refugees live and interact with other people and institutions, and their social, economic, cultural, environmental and material characteristics. Place is also a domain of refugee governance where political processes and bureaucratic infrastructures determine pathways to education, employment and welfare, and shape the daily lives of refugees in ways that may have significant mental health effects.

### 1.1 Mental health and place in post-migration context

Epidemiological studies show refugees are more likely than the general population to suffer from anxiety, PTSD, psychotic illnesses and major depression (Blackmore et al., 2020; Henkelmann et al., 2020; Nesterko et al., 2020). Typically, studies reporting pre-migration experiences of adversity and trauma show associations with PTSD and depression (Priebe et al., 2016; Schweitzer et al., 2011), whereas studies investigating post-migration factors show associations with anxiety, mood, substance-use disorders and psychosis (Harris et al., 2019; Hynie, 2018; Porter and Haslam, 2005). The role of pre-migration trauma and adverse experiences of flight in refugee mental health outcomes is well established (Fazel et al., 2005; Silove, 1999). However, studies have shown that the psychological distress of a precarious existence continues once settled in host countries (Porter and Haslam, 2005; von Werthern et al., 2018; Watters, 2001).

Refugees in Global North countries encounter post-migration factors which can play a key role in mental health outcomes (Blackmore et al., 2020; Fazel et al., 2005; Walker et al., 2020). Following an often prolonged and distressing asylum application process, refugees face a new set of challenges related to their status (Rowley et al., 2020; Walker et al., 2020). They encounter immediate everyday challenges related to social and economic determinants of health, including substandard housing conditions, unemployment or occupational downgrading, access and language barriers to healthcare, social isolation, racism and discrimination (Carter and Osborne, 2009; Hynie, 2018; Lumley-Sapanski, 2021; Mangrio and Sjögren Forss, 2017). Anti-immigration policies of deterrence, such as the UK's “hostile environment”, make access to basic care and opportunities very difficult despite their being entitled to such support by law (Asif and Kienzler, 2022; Goodfellow, 2020). Refugees must navigate an evolving set of structural and bureaucratic difficulties that present risks of marginalisation and poverty with implications for mental health (Abdelhady et al., 2020; Allsopp et al., 2014; Fassin, 2011).

Attention to the post-migration context has figured in refugee mental health research since the 1980s when dominant ‘stresses of acculturation’ models emphasised the role of cultural difference and adjustment difficulties in unfamiliar environments (McSpadden, 1987; Nguyen, 1982; Nicassio and Pate, 1984; Westermeyer et al., 1984). Most early studies acknowledged the lasting negative impacts of psychological trauma suffered by refugees and argued for specialised mental health interventions. Yet, their focus on the role of structural and environmental conditions remained limited (Clinton-Davis and Fassil, 1992; Muecke, 1992; Summerfield, 1991). Contemporary literature departs from these studies, focusing on notions of home-making (Brun and Fábos, 2015; Wimark, 2021), place-attachment (Albers et al., 2021), diaspora and community networks (Muir and Gannon, 2016; Orjuela, 2020), and neighbourhoods as both spaces of inclusion and exclusion (McWilliams and Bonet, 2015; Spicer, 2008).

According to some scholars, after the 2015–16 ‘refugee crisis’, we now see the issue of refugee integration come to the fore in the Global North countries, presenting a need to examine the role of host societies (Phillimore, 2020). At the same time, the war in Ukraine and reception of refugees from the region has highlighted stark differences in how people from diverse ethnic backgrounds and countries of origin are received and treated (De Coninck, 2022). Thus, links between refugee status and opportunities to tap into social and economic resources will likely be different depending on the situatedness of individual refugees in relation to their national and ethnic background, economic situation, gender and education levels. Likewise, ways in which refugees exercise their agency are varied and interrelated with post-migration contexts. For some, extensive diaspora connections can enable onward migration to be close to family and friends (Valentine et al., 2009; van Liempt, 2011), while remittances and other commitments and cultural practices can influence decisions about employment and housing (Lindley, 2009; Skovgaard Nielsen et al., 2015). Some dispersal areas have become refugee diaspora nodes because of permanent settlement there and expanding socio-cultural support networks (Kearns and Whitley, 2015; Sim, 2015).

Over 60% of the total refugee population live in urban areas; this proportion is likely even higher in countries of the Global North (Koizumi and Hoffstaedter, 2015; World Refugee Council, 2018). Urban mental health is subject to complex interactions between living conditions and environment, human biology, and psychosocial factors (Fitzgerald et al., 2016; Schofield and Das-Munshi, 2017). Studies show that neighbourhood ethnic density and urbanicity, i.e. population density, explain some of the increased risk of psychosis for migrants (Bécares et al., 2012; Schofield et al., 2017). In the general population, neighbourhood factors such as deprivation, urbanicity and low social cohesion are consistently linked with poor mental health (Allardyce et al., 2005; Kirkbride et al., 2014). However, there is little systematic evidence on the link between refugee mental health and, and few investigations of place-specific factors in the post-migration context for this population.

This review explores how post-migration contexts in refugee mental health have been approached in social science studies. Specifically, we identify, analyse and describe research results connecting place and refugee mental health in countries of the Global North and pay particular attention to methods and framings through which this link is established. We use a scoping review methodology to address the following research question: What are the main characteristics and pathways linking place to refugee mental health in Global North post-migration contexts, and what theoretical and methodological approaches are used to study this link? Following a broad typology of scoping review purposes outlined by Munn et al. (2018), our objectives are: 1) to review how research has been conducted on the relation between place and refugee mental health; 2) to explore available evidence on key characteristics or factors related to place in the selected studies; and 3) to identify and highlight gaps in the existing literature.

In the following section, we provide an overview of our literature search. Then, we provide a descriptive summary of the studies to highlight how place and refugee mental health have been studied jointly in research. Finally, we identify five key themes and briefly consider implications of our findings for refugee mental health policy and future research.

## 2 Methods

### 2.1 Search strategy and study selection

This review includes peer-reviewed qualitative, quantitative and mixed-methods original research studies from Global North countries. The following online databases were searched: PsycINFO, MEDLINE, Global Health, and Embase (via Ovid); PubMed and Sociological Abstracts. We also identified relevant literature through reference lists of included articles. The search strategy covered three broad categories: (i) refugees; (ii) mental health; and (iii) place (Table 1). The search was conducted in April 2021, with a follow-up search in January 2022 to scope newly published papers. Before the structured search, a precursory inquiry was undertaken using broad terms (e.g. refugee* AND mental*; refugee* AND *neighbourhood* OR neighbourhood; refugee* AND place* OR environment*) to identify key literature on refugee mental health in post-migration contexts. After familiarisation with key literature and debates, we narrowed our search down to empirical studies published in Global North countries between 2000 and 2021.

**Table 1:** Complete Ovid search strategy used.

Construct	Search terms
Refugees	refugee* OR forced migra* OR displace*
Mental Health	mental health OR mental disorder* OR anxiety OR psycho* OR depress* OR trauma* OR PTSD OR posttraumatic stress disorder OR post-traumatic stress disorder OR stress OR distress OR emotional stress* OR wellbeing OR well-being OR common mental disorder* OR CMD OR mental ill health OR mental illness* OR mental distress* OR neuropsychiatric* OR psychiatric* OR psychic* OR psychologic* OR psychopatho*
Place	neighbourhood* OR neighbourhood* OR urban* OR rural* OR city OR environment* OR place* OR resident*

Papers were screened against a set of pre-specified inclusion criteria, removing those which failed to elaborate on place-related factors or characteristics, or focus on:

● the refugee post-migration context in Global North countries
● refugee participants
● mental health.

We also excluded articles which were not:

● research papers
● peer-reviewed
● published in English.

**Fig. 1:**
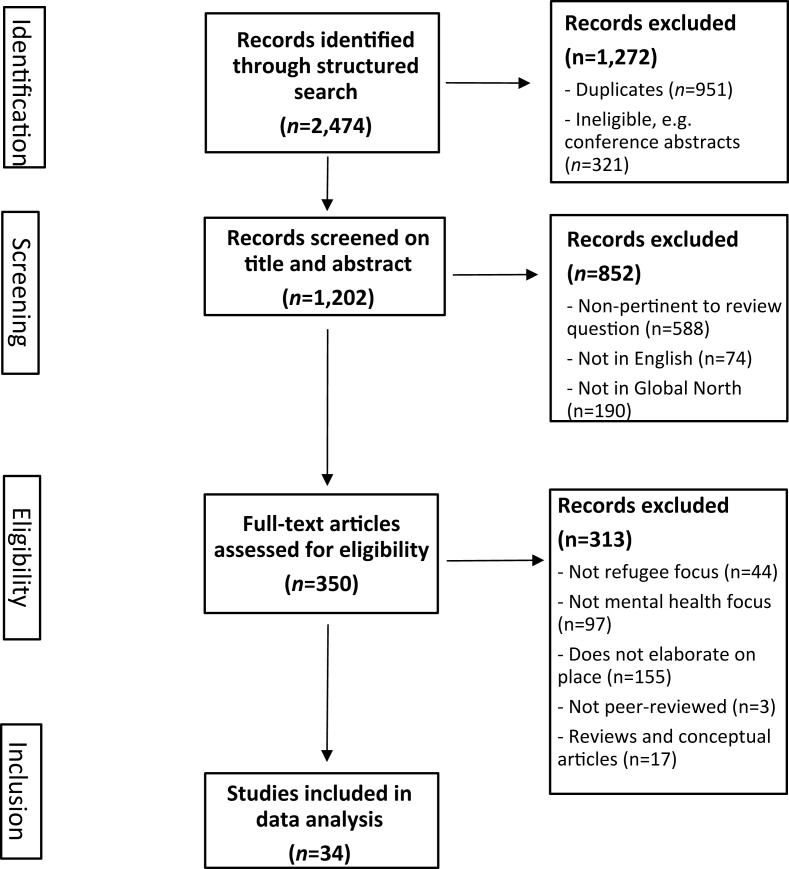
Adapted PRISMA Flow chart of the search strategy and results.

Fig. 1 summarises the selection process. Selection happened in two stages, initial screening of abstract and titles, then full-text screening. Broadly, all studies include information about places where refugee participants lived or had been approached by the authors. Specifically, we included articles that elaborated on place measures or characteristics (e.g., as independent variables or sampling criteria) and if place-related factors figured prominently in analysis or discussion and proved relevant to explanations and interpretations of mental health outcomes. Some articles included may not explicitly set out to examine how place relates to mental health but offer enough empirical material and insight to extract or derive its relevance based on constitutive factors of place analysed or discussed. Likewise, we adopted a broad conception of mental health and included articles that discussed outcomes such as mental disorders and psychological or emotional wellbeing in relation to where refugees lived.

### 2.2 Data extraction and analysis

To meet our objectives, we extracted study citation, sample and location, place definitions, place measures, primary mental health outcomes, data analysis strategy, and key findings (Peters et al., 2015). See Table 5. The final analysis was undertaken following a recommended two stage process (Arksey and O' Malley, 2005; Levac et al., 2010). Firstly, information based on extracted core data was synthesised into a descriptive summary of studies. Secondly, to address our research question, we adopted a narrative approach and organised the literature into main themes. We focused on exploration and description of how place and its characteristics in relation to refugee mental health are discussed in the literature.

## 3 Results

### 3.1 Descriptive information on studies

The review includes 34 studies: 14 quantitative, 14 qualitative and 6 mixed-methods. All qualitative and most quantitative studies consisted of original research based on the collection of primary data (82%) (see Table 2). Six quantitative studies utilised large data sets for secondary data analysis. These included: the Norwegian Patient Registry (Finnvold and Ugreninov, 2018), the National Latino and Asian American Study (NLAAS) (Kim, 2016), the New Canadian Children and Youth Study (NCCYS) (Beiser and Hou, 2016), and the GoWell survey in Scotland (Kearns et al., 2017). Two studies used data from the Somali Youth Longitudinal Study in the US (Gillespie et al., 2020; Lincoln et al., 2021).

**Table 2:** Descriptive information of studies.

Variable	Number of studies	Percentage of studies (*n = 34*)
**Study type**		
Original research study	28	82%
Secondary data analysis	6	18%
Study design		
Qualitative study	14	41%
Psychometric assessment (cross-sectional)	9	26%
Cross-sectional (incl. mixed methods)	4	12%
Psychometric assessment (longitudinal)	3	9%
Cohort study	2	6%
Intervention study	2	6%

Most qualitative studies used interviews and focus groups (*n* = 9). Some used approaches such as: sensory ethnography with refugees involved in gardening activities (Biglin, 2020), creative mapping and interviews (Brenman, 2020; Sampson and Gifford, 2010), participatory research, workshops and interviews (ASPIRE) (Murray et al., 2019), and ethnographic fieldwork (Grønseth, 2001).

The most represented refugee groups were Somalis (*n* = 10) and Syrians (*n* = 7), followed by Afghans, Bosnian and Iraqis (*n* = 4). Then Vietnamese, Ethiopian, Tamil, Iranian and Eritreans (*n* = 3), and El-Salvadoran, Kenyan, Croatian, Serbian, Mexican and Sudanese refugees appear once. Fourteen studies did not specify ethnicity or country of origin but indicated regions, such as the Middle East, Africa, and Asia. Most studies focused on adults (*n* = 30), four focused on children (<18 years). Study locations were mostly the UK and Australia (*n* = 6), followed by the US (*n* = 4), Canada (*n* = 3), Norway, Germany and Sweden (*n* = 2), and Denmark (*n* = 1). A few studied cross-country comparisons: UK/US (*n* = 1), Norway/Lebanon (*n* = 1), Sweden/Turkey (*n* = 1), Norway/Denmark (*n* = 1), and Canada/US (*n* = 3).

### 3.2 Locations and characteristics of place

Places where research was conducted varied (see Table 3). Most studies occurred in urban locations (*n* = 15) and referred to broad place categories e.g., city or urban centres. Some studies discussed micro-scale locations (*n* = 5) like accommodation, schools and urban allotment; rural locations (*n* = 4); and countries (*n* = 4; including cross-country comparison). A few compared urban and rural areas (*n* = 3) and explored lived experiences in different neighbourhoods (*n* = 3).

**Table 3:** Types of places.

Type of place	Number of studies	Percentage of studies (*n = 34*)
City/urban area	15	44%
Micro-scale locations	5	15%
Country	4	12%
Rural/regional area	4	12%
Urban and rural comparison	3	9%
Neighbourhood	3	9%

Most studies identified multiple intersecting characteristics of place considered relevant for refugee mental health outcomes. Table 4 shows characteristics primarily, but not exclusively, discussed in the referenced studies. Additionally, access to green spaces and outdoor physical activities (Biglin, 2020); material and social aspects of institutions (Brenman, 2020; Leiler et al., 2019; Zehetmair et al., 2021); built and natural environment, local history and culture (El-Bialy and Mulay, 2015; Grønseth, 2001; Herslund and Paulgaard, 2021); and ethnic density (Finnvoqld and Ugreninov, 2018) were included as important place characteristics relevant to refugee mental health.

**Table 4:** Key characteristics of place.

Characteristics of place	Source
Neighbourhood safety, deprivation, or violence	Beiser and Hou (2016); Gillespie et al., (2020); Kim (2016); Liu et al. (2020); Montgomery, 2008; Salhi et al., (2021); Warfa et al. (2006); Ziersch et al. (2017)
Employment and education issues	Blight et al. (2006); Correa-Velez et al. (2011); Fozdar (2009); Papadopoulos et al. (2004); Warfa et al. (2012); Ziersch et al. (2020)
Social environment and discrimination or stigma	Bhui et al. (2012); Elswick et al. (2021); Glorius et al. (2020); Lincoln et al. (2021); Murray et al. (2019)
Safe or substandard accommodation or housing	Cheung Chung et al. (2018); Kearns et al. (2017); Miller et al. (2002); Sampson and Gifford (2010)
Refugee governance, healthcare, and support services	Beza et al. (2021); Goodkind et al. (2014); Haj-Younes et al. (2020)

**Table 5:** Mental health and psychosocial categories across quantitative and mixed methods studies.

Variable	Number of studies	Percentage of outcomes (*n* = *34*)
Quality of life	7	19%
Depression	6	17%
PTSD/post-traumatic symptoms	6	17%
Anxiety	5	14%
Subjective wellbeing/mental health	4	11%
General psychiatric morbidity	3	8%
Psychological/emotional distress	2	6%
Emotional problems/aggressive behaviour	2	6%
Admission to mental health institutions	1	2%

Note. Listed variables represent broader categories of investigated mental health outcomes. A higher number of studies is reported as some studies had more than one mental health or psychosocial outcome.

### 3.3 Conceptions of mental health

In quantitative and mixed methods studies, the most common mental health conditions investigated included: depression (n = 6), PTSD (n = 6), anxiety (n = 5), and subjective wellbeing (n = 4) (see Table 5). Quality of life was explored in seven studies. Mental health and quality of life were investigated with various measures. The most commonly used mental health measures in quantitative studies were HTQ (Harvard Trauma Questionnaire) (*n* = 5); HSCL-25 (The Hopkins Symptom Checklist-25) (*n* = 3); and WHOQOL-BREF (World Health Organisation Quality-of-Life Scale) (*n* = 3).

Qualitative studies addressed refugee mental health in different ways. Three explored refugee place-making, healing and wellbeing practices through the concept of therapeutic landscapes broadly conceptualised as places “where the environment and human perception interact and produce a therapeutic atmosphere” (Biglin, 2020, p. 1; El-Bialy and Mulay; 2015; Sampson and Gifford, 2010). A few studies explored sense of wellbeing as both subjective experience and an individual psychological condition (Glorius et al., 2020; Grønseth, 2001; Ziersch et al., 2020, 2017); resiliency and role of “environments to facilitate the navigations of individuals for the resources they need to cope with adversity” (Liu et al., 2020, p. 2); experiences of psychosocial support and mental healthcare (Brenman, 2020; Zehetmair et al., 2021). In addition, some unpacked feelings of stress and insecurity (Herslund and Paulgaard, 2021); understandings of depression and psychological distress in relation to settlement experiences (Fozdar, 2009); nervousness, insomnia, loss of appetite and social isolation (Miller et al., 2002); geographic isolation and experiences of family violence (Murray et al., 2019); loss of social cohesion and self-reported psychological distress, worry, and anxiety (Warfa et al., 2006). Further details on conceptions and measures of mental health use by each study are highlighted in Table 6 below.

**Table 6:** Descriptive summary of included studies.

Source	Description	Setting	Study Design	Sample	Place definition/location	Place characteristics or measures	Primary mental health outcomes	Key findings
Beiser and Hou (2016)	A cohort study based on a survey on emotional problems and aggressive behaviour in refugee and immigrant youth living in six major urban areas in Canada.	Canada	Quantitative; cohort	Refugee and immigrant children ages 11-13 (n = 90)	Neighbourhoods in major urban areas.	Discrimination; poor quality of neighbourhood; people selling/using drugs; alcoholics and a lot of drinking; young people causing trouble; ethnic/religious tension.	Self-reported emotional problems and aggressive behaviour	Refugee youth had overall higher emotional problems and aggressive behaviour levels than immigrant youth from the same countries. They also reported higher levels of experienced discrimination. Refugee mothers were less critical of neighbourhood quality than immigrant mothers.
Beza et al. (2021)	A comparative survey of Syrian refugees residing in a refugee camp and urban apartments in Northern Greece to compare health-related quality-of-life measures comprising participant perceptions of their physical and mental health.	Greece	Quantitative; cross-sectional	Refugee adults (n = 161)	A refugee accommodation site and urban apartments.	Governmental and non-governmental programs aimed at accommodation support in urban settings; living conditions.	The Mental Component Scale and the Physical Component Scale and of the health-related quality-of-life (HRQoL).	Refugees and asylum seekers living in accommodation site reported lower physical and mental health scores. Refugees and asylum seekers living in urban apartments had slightly better mental health outcomes compared to camp residents. Urban environments may offer increased sense of safety, resemble home, and satisfy everyday needs. Refugees in urban apartments received better support and had better living conditions.
Bhui et al. (2012)	Survey and questionnaire with Somali refugees in London about post-migration residential mobility, social support, and their impact on psychiatric disorders.	United Kingdom	Quantitative; cross-sectional	Refugee adults (n = 142)	Urban area; postcode area and electoral ward.	Index of Multiple Deprivation (IMD) based on housing, education, health, and child deprivation; social support; discrimination.	Psychiatric disorders (MINI)	Psychiatric disorders are more common among Somali refugees who have moved accommodation within five years of arrival in the UK. Social support networks safeguard against psychiatric morbidity related to mobility. For many, residential mobility takes place to seek out social networks and to further employment prospects. People with mental disorders are less likely to move to better places and move more often because of the stigma and unstable housing. Residential mobility need not be associated with high risks of psychiatric disorder for those in good support networks and where choice of the move is maximised.
Biglin (2020)	Sensory ethnography with refugees participating in the urban allotment in the North-West UK, focusing on therapeutic effects of communal gardening for mental health and wellbeing.	United Kingdom	Qualitative	Refugee adults (n = 8)	Urban allotment.	Green space; silence; physical activities.	Therapeutic and healing experience; stress reduction.	The allotment was conceptualised as therapeutic landscape and offered refugees therapeutic interactions with the natural environment and each other. The embodied experience of presence alleviated feelings of loneliness and addressed distressful memories and experiences of displacement. Therapeutic experience was achieved through embodiment rather than verbal communication.
Blight et al. (2006)	Survey of refugees from Bosnia-Herzegovina about residential mobility, employment status and mental health in two urban and rural Swedish regions with high and low employment levels among refugees, respectively.	Sweden	Quantitative; cross-sectional	Refugee adults (n = 360)	Urban and rural regions.	Employment levels among refugees.	Mental health status (Göteborg Quality of Life)	Living in the urban region statistically significantly increased the odds of worse mental health status for women but not men. For men, being unemployed showed a statistically significant increase in worse mental health status but did not for women. People in rural region with higher employment levels moved more often to seek employment/better job than people in urban region with lower employment levels. No association was found between moving to employment and better mental health outcomes compared to not moving or moving for other reasons.
Brenman (2020)	Qualitative study of refugee service users, at an intercultural psychotherapy centre in London, UK, accessed and staffed by refugees and other migrants.	United Kingdom	Qualitative	Refugee adults (n = 2)	Psychotherapy centre.	Material and spatial aspects of the premises; unstable funding.	Experiences of access to mental health care and sense of belonging.	Socio-political context of UK voluntary mental health sector and austerity measures shape geographical and spatial characteristics of the psychotherapy centre. Lack of funding and of permanent location made it difficult for the centre to create welcoming environment. In turn, this created a sense of 'precarious belonging' among service users - characterised by ambivalence about their position as clients, uncomfortable waiting room experiences; and uncertainty about being deserving of care. Experience of belonging and sense of precarity are produced in situated relations between people, space and materiality.
Cheung Chung et al. (2018)	Comparative study of psychological distress in Syrian refugees residing in Sweden and Turkey.	Sweden and Turkey	Quantitative; cross-sectional	Refugee adults (n = 1197)	Countries.	Living conditions; safe accommodation and access to community centres in a city (Sweden); substandard shelters in remote rural area near the Syrian border (Turkey).	Post-traumatic stress symptoms (HTQ) and psychiatric co-morbidity (GHQ-28).	Location of residence significantly correlated with post-traumatic stress symptoms and psychiatric co-morbidity, and co-varied with trauma exposure characteristics and depression. Respondents in Turkey had significantly higher rates than in Sweden. Living conditions in Turkey were considerably worse than in Sweden in terms of facilities, shelter, basic infrastructure. Refugees in Sweden lived in safe accommodation and received government welfare support and had access to amenities.
Correa-Velez et al. (2011)	Survey comparing the health status and use of health services among recently arrived men with refugee backgrounds living in urban and regional areas of South-East Queensland.	Australia	Quantitative; cross-sectional	Adult refugees (n = 233)	Urban and regional areas.	Limited access to education and employment; limited access to affordable medical care; poor provision of language services; experiences of discrimination.	Anxiety and depression (HSCL-25), post-traumatic stress symptoms (HTQ); subjective well-being (WHOQOL-BREF).	Men living in regional areas report poorer levels of well-being in the environment domain and are more likely to visit hospital emergency departments. They also are more likely to report negative experiences at educational institutions, take jobs below their skill levels, show job dissatisfaction, and report greater discrimination and difficulties in securing employment. Refugee men living in urban areas are more likely to have a long-standing illness or disability and report poorer health status than those settled in regional areas.
El-Bialy and Mulay (2015)	Qualitative study of resettled 'former' refugees in the city of St.John in Newfoundland and Labrador, focusing on the impact of place-related determinants on the sense of wellbeing.	Canada	Qualitative	Adult ‘former’ refugees (n = 10)	Small urban centre.	Population (n = 200,000); built environment; harsh natural environment; local history and culture; and low ethnic diversity.	Sense of wellbeing; experiences of coping, support and challenges.	Place-related social and environmental determinants of refugee wellbeing have ambivalent effects - they present as supports and/or challenges and their presentation can change over time.
Elswick et al. (2021)	A study of trauma intervention and after school support program among African refugee children in urban West Tennessee.	US	Intervention study; mixed methods	Refugee children (n = 88)	Large urban area.	High overall and child poverty; below national average population health; health and mental health care shortage.	Symptoms of PTSD and depression (CPSS); emotional distress (SUDS).	The after-school support programme that was culturally adapted for a specific group of resettled African refugee children in urban West Tennessee involved drumming sessions and peer-based pyramid mentoring. Results showed clinically significant improvement in participant reported PTSD symptomology from pre to post assessment, and gradual improvement in children's distress or emotional state. The inclusive, culturally informed environment during regular after school sessions alleviated otherwise highly disadvantageous circumstances of refugee social and material environment.
Finnvold & Ugreninov (2018)	Health records register analysis of refugees in Norway focusing on relation between ethnic density and risk of being hospitalised in mental healthcare institution and the time spent there.	Norway	Quantitative; cross-sectional	Refugee adults (n = 30871)	Ethnic enclave; immigrant residential clusters; neighbourhood.	Ethnic density; neighbourhood deprivation (level-of-living conditions).	Admissions to mental health hospitals and time spent there.	Refugees who live in 'ethnic enclaves' appear to have lower probability of being admitted to mental healthcare institution and spend less days there if they are admitted. Neighbourhood deprivation did not increase the risk of the admission. Ethnic density appears to be a protective factor for refugee mental health.
Fozdar (2009)	A study based on interviews, questionnaires, and focus groups with African and ex-Yugoslav refugees in Perth, Western Australia, focusing on relation between employment, social integration, and depression.	Australia	Qualitative	Refugee adults (n = 200)	City	Unemployment; occupational downgrading; discrimination; structural barriers to gainful employment.	Depression; psychological distress.	Both refugee groups experienced issues with finding employment and occupational downgrading. Although, both groups did not readily subscribe to biomedical model of depression, they revealed that employment issues along with discrimination and stigma underpinning these, caused psychological distress and depression. Another causal factor for depression was social isolation and loss of social life associated with one's own cultural identity and community ties.
Gillespie et al. (2020)	A study of residential mobility and neighbourhood violence effects on mental health among Somali young adults living in urban areas in United States and Canada.	US and Canada	Quantitative; longitudinal	Refugee adults (n = 198)	City	Discrimination; housing instability; interpersonal conflict; neighbourhood violence**.**	Post-traumatic stress symptoms (HTQ)	Experiences of interpersonal violence, discrimination, financial insecurity affect housing mobility and in/stability in both regions. Neighbourhood safety and violence is common reason for moving. Access to stable and safe housing was associated with changes in symptoms of posttraumatic stress. There were significant differences in rates of moving by region, and between voluntary and forced movers. Participants who experienced a forced move in the past year reported more severe symptoms a year later, while those who moved voluntarily reported fewer symptoms.
Glorius et al. (2020)	A study of 32 municipalities in rural Germany comprising a survey among rural residents and a series of qualitative interviews with refugees, focusing on social contact, integration and its relation to refugee wellbeing and place attachment.	Germany	Mixed methods; cross-sectional	Refugee adults (n = 139) and local residents (n = 908)	Municipalities from the districts classified as "very rural"; neighbourhood defined as "your ultimate living environment"	Population (n < 20,000); everyday encounters and social relationships in neighbourhoods; expectations, perceptions and experiences of relationships and social contact among resident population and refugees.	Wellbeing and place attachment.	Positive social relationships are of key importance for refugee experience of wellbeing and place attachment. Greater social contact between local residents and refugees leads to more prevalent positive relationships. Less contact increased expression of negative stereotypes toward refugees. Intensity of weak social interactions can grow and develop into stronger positive ties and place attachments. Social ties can act as protective mechanisms for new refugees to overcome sense of otherness and non-belonging.
Goodkind et al. (2014)	A study of community-based advocacy and learning intervention to address social determinants of mental health among African refugees living in a large city in New Mexico, US.	US	Intervention study; mixed methods; longitudinal	Refugee adults (n = 36)	Large city.	Quality of life; access to resources.	Psychological wellbeing.	The intervention involved refugees and undergraduates working together on two intervention components, learning and advocacy. Both components addressed social determinants of mental health experienced by participants living in the city. Results showed intervention success in acquisition of new skills and knowledge; increased environmental mastery, self-sufficiency, and self-confidence; and decreased feelings of discrimination, among others. The quantitative and qualitative data suggest that the intervention had positive impacts on refugee's mental health and wellbeing.
Grønseth (2001)	An ethnographic study of Tamil refugees in small fishing villages in the far north of Norway, focusing on relations between social context, settlement and refugee health and wellbeing.	Norway	Qualitative	Refugee adults (n = N/A)	Small fishing village.	Small population; cold climate; generous Norwegian welfare state; physical safety and security.	Wellbeing and health.	Refugees remained distant from the local communities and formed their own communities and associations. Younger generation refugees were more involved in local life and planned to stay in Norway in contrast to older ones. On the one hand, refugees valued personal and economic safety, healthcare, and employment opportunities available in remote villages. On the other, they felt segregated as refugees, culturally different and did not feel that health professionals understood them. Healthcare interactions revolved around management of 'cultural differences.' Refugee's experience of wellbeing was associated with intra- and inter-community differences.
Haj-Younes et al. (2020)	A prospective longitudinal study of quality of life among Syrian refugees resettled from Lebanon to Norway	Norway and Lebanon	Quantitative; longitudinal	Refugee adults (n = 353)	Countries.	Living conditions; physical safety and security; quality of health and social care.	Quality of life (WHOQOL-BREF)	Quality of life outcomes increased significantly in the short follow-up period of one year. The pre-arrival scores for physical health, psychological health, and environment were rated significantly lower than the mean scores from the WHOQOL-BREF international field trials. While the physical and psychological domains improved significantly after resettlement, they remained lower than international reference scores. Whereas in the environmental domain, the mean scores surpassed the levels of international reference scores.
Herslund and Paulgaard (2021)	A study of refugees resettled in rural areas of Denmark and Norway, focusing on the bodily and sensory experiences of daily life, stress, and wellbeing.	Norway and Denmark	Qualitative	Refugee adults (n = N/A)	Rural areas in two countries.	Physical environment; climate; living conditions.	Stress and wellbeing.	Refugees dispersed to rural Danish and Norwegian locations experienced them as disorienting, especially if they had lived in urban areas before resettlement. The harsh climate, weather and short daylight was stressful. These negative experiences were offset by social connections with other refugees and local organisations.
Kearns et al. (2017)	Community survey of British-born respondents, social and economic migrants, and refugees and asylum seekers, measuring four health outcomes, including mental health in the context of life in Glasgow, Scotland.	United Kingdom	Quantitative; cross-sectional	Refugee adults (n = 692); migrants (n = 712); British-born (n = 5787)	Post-industrial city.	High levels of deprivation; poor-quality housing; run-down environments.	Physical and mental health (SF-12v2); mental well-being (WEMWBS).	Refugee health declined with more time spent awaiting asylum decision but showed improvement after a leave-to-remain decision. Asylum seekers who received temporary leave to remain ('cessation clause') reported continued deteriorating health. Refugees and asylum seekers who had lived in a deprived area for more than a year had slightly better self-rated health and well-being than recent arrivals and indigenous and social and economic migrants.
Kim (2016)	The study based on US resettled refugee subsample from the 2002–2003 National Latino and Asian American Study (NLAAS) data from the Collaborative Psychiatric Epidemiology Surveys, investigating social determinants of mental health.	US	Quantitative; cross-sectional	Refugee adults (n = 656)	Neighbourhood environment.	Social cohesion and physical safety of the neighbourhood.	Self-rated mental health (SRMH), mood disorders, and anxiety disorders.	Neighbourhood environment was associated with self-rated mental health but was not associated with mood and anxiety disorders.
Leiler et al. (2019)	Study of refugees and asylum seekers in refugee housing facilities in Sweden focusing on housing facility and prolonged asylum waiting time relevance for mental health outcomes.	Sweden	Quantitative; cross-sectional	Refugee adults (n = 143) and asylum seekers (n = 367)	Refugee housing facilities.	High levels of uncertainty and unpredictable conditions regarding housing location; limited health care; high levels of passivity; low levels of meaningful daily activities.	Depressive symptoms (PHQ-9), symptoms of anxiety (GAD-7), risk of having post-traumatic stress disorder (PC-PTSD), and quality of life (WHOQOL-BREF).	Refugees with residence permits showed better mental health outcomes than asylum seekers but had worse outcomes than those resettled in permanent housing. Quality of life was rated below population norms and showed negative correlation with mental health outcomes. Due to unavailable housing, many refugees stayed in facilities after receiving their residence permits. Prolonged asylum decision times and staying in refugee housing facilities contribute to worse mental health outcomes in refugees.
Lincoln et al. (2021)	Study of how individual and collective-level factors mediate associations between discrimination and mental health among Somali youth (n = 439) in four urban locations in North America.	US and Canada	Quantitative; cross-sectional	Refugee young adults (n = 439)	Four urban centres.	Discrimination; neighbourhood social cohesion; intergenerational relations.	Anxiety; depression (HSCL-25); post-traumatic stress symptoms (HTQ).	Experiences of discrimination had a direct effect on worsening symptoms of all mental health outcomes. Neighbourhood collective efficacy which measured social cohesion and intergenerational closure was not related to discrimination or mental health.
Liu et al. (2020)	A study of resiliency factors among recent (less than 5 years) and settled (more than 5 years) refugees and resettlement professionals in Vancouver, Canada.	Canada	Qualitative	Refugee adults (n = 21) and professionals (n = 13)	City and metropolitan area.	Neighbourhood safety; local amenities; living environment.	Resiliency and wellbeing.	Living environment was identified as an external resiliency factor. Access to natural spaces and neighbourhood safety, particularly for women, improved sense of wellbeing. Access to familiar food and having freedom to practice own religion helped refugees feel accepted. This contributed to a sense of living in multicultural society, with opportunities to learn about other cultures, and increased integration into wider society.
Miller et al. (2002)	Narrative study of 28 adult Bosnian refugees in Chicago, focusing on exile-related stressors with implications for mental health.	US	Qualitative	Refugee adults (n = 28)	City	Poverty; high cost of rent; inadequate housing and overcrowding; new language and environment.	Nervousness; insomnia; loss of appetite; loneliness.	Insufficient income made safe and adequate housing unaffordable, and with lack of other basic necessities, was the single most common source of post-migration distress. Participants felt social isolation and loneliness in stark contrast to their past lives filled with friends and family members. For some, being alone gave an opportunity to distance from others and avoid painful conversations. Environmental mastery was dependent on becoming familiar with local life, city infrastructure and language. English as second-language classes were difficult for people who were traumatised and had issues with memorising.
Montgomery (2008)	A study of young refugees from Middle Eastern countries who arrived in Denmark in early 1990s with follow up in 2000-01 looking at the influence of traumatic experiences before emigration and post-migration social life on mental health.	Denmark	Quantitative; cohort study	Refugee children (n = 131)	Country	Present social situation; residential mobility; and stressful experiences.	Externalising and internalising behavioural problems (YSR & YASR).	Externalising behaviours were predicted by witnessing violence in Denmark, such as attacks on others, and a higher number of schools attended. Internalising experiences were predicted by traumatic experiences before arrival, stressful experiences after arrival and experiences of discrimination.
Murray et al. (2019)	A study of refugee and immigrant women who had experienced family violence post-migration and with service providers, comparing eight urban and remote sites in Victoria and Tasmania, Australia	Australia	Qualitative	Refugee adults (n = 46)	Inner-city and outer metropolitan, and regional/remote sites.	Geographic isolation; availability and access of specialised services; local support.	Experiences of gender-based violence; help-seeking; use of services.	Place-based differences determine the quality of support for refugee women who experience gender-based violence. Factors such as proximity to metropolitan centres may increase the availability of culturally informed services, translators but also have less accessibility due to higher demand. Relocating to remote support centres allow for longer shelter periods and an escape from community stigma but increase exposure to local discrimination.
Papadopoulos et al. (2004)	A study of Ethiopian refugees about their experiences of settlement in the large metropolitan areas in the UK and perceptions of health, mental health, and wellbeing.	United Kingdom	Mixed methods; cross-sectional	Refugee adults (n = 98)	Country; urban areas.	Housing and living conditions; legal status; employment options.	Experiences of stress, sadness, and depression; mental wellbeing.	Many participants reported stressful living and housing conditions. Several socio-economic factors, such as lack of money, housing problems, unemployment, and pollution were reported as causes of ill health. Having a permanent refugee status (indefinite leave to remain) was associated with better employment situation, although many reported working below their skill level. Some of the participants reported having suffered from depression or mental illness since coming to the UK. Of these the majority were men half of whom were employed. Other stressful factors included social isolation, uncertainty of asylum decision, problems with English language and lack of spiritual life. Almost half of respondents reported to feel sad or unhappy for long periods of time.
Salhi et al. (2021)	Survey of Somali refugees in five cities in the US and Canada focusing on relationship between pre- and post-resettlement experiences of violence and mental health.	US and Canada	Quantitative; longitudinal	Refugee adults (n = 383)	Cities.	Exposure to violence.	Symptoms of depression and anxiety (HSCL-25); post-traumatic stress symptoms (HTQ).	Refugees in the US and Canada are commonly exposed to post-resettlement violence which is more associated with poor mental health outcomes than exposure to pre-resettlement violence. In the post-resettlement context, the types of violence that refugees are exposed to are directly related to resettlement areas with usually high levels of poverty and community violence, such as shootings and physical attacks.
Sampson and Gifford (2010)	A study of young refugees in Melbourne, Australia about the role of place in supporting the health and well-being during settlement.	Australia	Qualitative	Refugee children (n = 120)	Local areas in outer suburbs of the city; English Language Schools.	Relatively affordable housing; middle to lower income households; new housing developments; and poor infrastructure including transport and recreation facilities.	Experiences of health, well-being, and healing.	Therapeutic landscapes for young refugees were made up of four types of places identified by participants as promoting experiences of health, restoration, and sense of wellbeing: places of opportunity, place of restoration, places of sociability and places of safety. These places entailed housing and home, recreational facilities, libraries, and school. Respondents emphasised such characteristics as safety, quiet, good neighbours and classmates, green and beautiful spaces. Both the physicality and the sociality of places are important for promoting homemaking and recovery from traumatic experiences.
Warfa et al. (2006)	A study with Somali professionals and lay Somalis in East and South London, UK, focusing on post-migration residential mobility, mental health, and service use.	United Kingdom	Qualitative	Refugee adults (n = 34)	Inner city areas.	Deprivation; safety; violence; racism.	Poor mental health; psychological distress.	Residential instability was driven by short-lets in temporary housing, poor housing conditions, overcrowding, racism and discrimination and employment opportunities. Frequent moves led to disrupted health and mental health service use because of different Primary Care Trust catchment areas, disruptions of children's schooling, and overall psychological distress caused by lack of control and the future. Respondents saw residential instability as bad for mental health and detrimental to social cohesion and general wellbeing. Respondents on lower incomes were more exposed to residential instability than professionals and at times moved into more deprived areas than before.
Warfa et al. (2012)	A study of refugees in two cities, London, UK and Minneapolis, US, exploring migration experiences, employment status and psychological distress.	United Kingdom and US	Mixed methods; cross-sectional	Refugee adults (n = 236)	Cities.	Legal status; employment; stigma.	Psychiatric disorders (MINI)	Compared with Minneapolis, respondents in London experienced more problems of family separation, legal uncertainties and were more likely to be unemployed than Minneapolis respondents. These differences appeared to predict different risks of mental disorders in the two cities. Refugees living in London were more likely to report major depression and any mental disorder. Employment had most of the impact by reducing the odds of major depression by a significant amount.
Zehetmair et al. (2021)	A study of the mother and child psychosocial support centre for refugee women located at a reception and registration centre in Germany.	Germany	Qualitative	Refugee adults (n = 16)	Psychosocial care centre; reception and registration centre.	Women-only; sheltered environment; language classes; childcare; socialising space.	Wellbeing and psychosocial support.	Psychosocial care offered to refugee women included German language classes, childcare support, socialising and leisure activities and were valued by women who attended the centre. Language classes were most valued as they provided women with essential skills for social integration. Most participants also appreciated the women-only concept due to their past experiences with gender-based violence, although some also expressed concern that men may feel excluded from support and that the concept did not correspond to German social norms about which they were learning.
Ziersch et al. (2017)	A study of refugees and asylum seekers in South Australia about relations between housing and wellbeing, health, and mental health.	Australia	Qualitative	Refugee adults (n = 28) and asylum seekers (n = 22)	Neighbourhood and housing.	Neighbourhood safety and disorder; social connections.	Mental health, health and wellbeing.	Neighbourhood safety and disorder may foster or undermine refugee experiences and perceptions of housing with implications for mental health and wellbeing. Affordability of housing, security of tenure and suitability in relation to physical aspects such as condition and layout were central for sense of wellbeing and mental health.
Ziersch et al. (2020)	A study of Southeast Asian and African refugees in rural town in South Australia, focusing on integration and social determinants of health and wellbeing.	Australia	Qualitative	Refugee adults (n = 44)	Rural town.	Population (n < 30,000); low cultural and linguistic diversity; education and median weekly income and occupational levels below the South Australian and Australian average.	Wellbeing and health.	Rural settings offered a strong sense of safety and some elements of social connectedness and support which enabled integration and health and wellbeing. Main challenges that cause stress and undermined sense of wellbeing included lack of secure employment, underemployment or work below skill levels, poor transportation and geographical isolation, experiences of discrimination and racism, and constrained access to services, including healthcare.

Abbreviations:HRQoL (Health-related Quality of Life), MINI (Mini International Neuropsychiatric Interview), HTQ (Harvard Trauma Questionnaire), GHQ-28 (28-item General Health Questionnaire), CPSS (Child PTSD Symptom Scale), SUDS (Subjective Units of Distress Scale), YSR (Youth Self Report), YASR (Young Adult Self Report), HSCL-25 (The Hopkins Symptom Checklist-25), WHOQOL-BREF (World Health Organisation Quality-of-Life Scale), SF-12v2 (Medical Outcomes Study Short-Form version 2), WEMWBS (Warwick-Edinburgh Mental Well-Being Scale), PHQ-9 (The Patient Health Questionnaire-9), GAD-7 (General Anxiety Disorder-7), PC-PTSD (The Primary Care PTSD Screen for DSM-5.

### 3.4 Main themes in the literature

#### 3.4.1 Theme 1: Material and physical characteristics of place can have therapeutic or harmful effects on refugee mental health

Several studies focussed on how physical and material elements of place affected refugees’ mental health and wellbeing. Three qualitative studies using the concept of therapeutic landscapes underscored how quiet, welcoming and reliably safe spaces in both urban areas and remote natural environments, provided resources to improve mental health and wellbeing (Biglin, 2020; El-Bialy and Mulay, 2015a; Sampson and Gifford, 2010a). In an ethnography of an urban allotment, Biglin (2020) explored place-making and the therapeutic aspects of gardening. The study shows how sharing outdoor space and tasks with others enabled an embodied experience of presence, reducing loneliness without verbal communication and turning nostalgic longing into connection rather than disconnection with their former life.

The concept of therapeutic landscapes in these studies elucidates active refugee involvement in finding pathways to healing. A study by El-Bialy and Mulay (2015) noted how harsh climate and remote natural environments challenge refugees who are used to different climes. Refugees in remote Canadian provinces described the climate as oppressive, saying lack of sunlight exacerbated homesickness and distress, even after living there for many years. However, the study also reported that some came to appreciate the wilderness and outdoor activities, saying it helped mitigate pre-migration trauma, homesickness and depression, and fostered a sense of wellbeing. Similar findings are reported by Herslund and Paulgaard (2021) for refugees dispersed to rural Danish and Norwegian locations. Likewise, the harsh weather and short daylight was found to be stressful but these experiences were offset by social connections with other refugees and local organisations.

Materiality and physicality of place can also be protective for refugee mental health and wellbeing. Sampson and Gifford (2010) found that the most important places for young, recently arrived, refugees in Australia were home, school, libraries and parks. This enabled exploration of new social/geographical environments which became associated with safety and belonging. The authors categorised them as places of opportunity, beauty and comfort, sociality and safety. Visual methods used in the study also revealed a contrast between protective and dangerous places. Places of safety were located near places associated with violence and threats. For example, train stations and secluded school grounds exposed participants to risks of attacks and racism. The protective role of place was also highlighted in a study by Liu et al. (2020) who identified a positive ‘living environment’ as ‘external resiliency factor’ that includes neighbourhood safety and access to natural spaces.

Underfunded and ill-equipped places undermine recovery even if their purpose is meant to be therapeutic. A study by Brenman (2020) showed how geographical and spatial instability of a psychotherapy centre undermined refugee mental health. Lack of funding and no permanent location hindered a welcoming and stable environment. Consequently, refugee service users felt a sense of 'precarious belonging', reporting a strong sense of ambivalence as clients and uncertainty about being deserving of care. Studies that explored material aspects consistently showed that substandard material and physical circumstances of refugee accommodation sites and temporary housing undermine recovery or make refugee mental health worse (Beza et al., 2021; Cheung Chung et al., 2018).

#### 3.4.2 Theme 2: Place-specific social determinants of refugee mental health

Studies highlighted that social determinants of refugee mental health are place-specific at both national and cross-national levels. Three studies using the WHOQOL-BREF found self-reported quality of life outcomes within the ‘environment domain’ differed both between resettlement countries, and within the same country, for instance between regional and urban areas (Correa-Velez et al., 2011; Haj-Younes et al., 2020; Leiler et al., 2019). In Australia, recently-arrived refugee men in regional areas reported better subjective health status but a poorer quality of life in the environmental domain compared to those in urban areas (Correa-Velez et al., 2011). According to the authors, better health status reports may reflect regional jobs requiring physically fit workers. However, employment opportunity appeared offset by occupational downgrading, greater discrimination and worse educational experiences, resulting in low quality of life outcomes.

Results from a study using a cross-national comparison of Syrian refugees resettled from Lebanon to Norway, showed quality of life increased significantly across physical, psychological and environment domains after one year (Haj-Younes et al., 2020). While the physical and psychological domains improved significantly after resettlement, overall, they remained lower than international reference scores. The environment domain had the lowest ratings at baseline but showed the highest improvement, surpassing the mean scores from the WHOQOL-BREF international field trials. The authors attributed this stark contrast to poor refugee conditions in Lebanon compared to Norway's advanced refugee support system and welfare provision. Underlining these location-specific differences, a study of refugees in Swedish migration agency housing facilities found clinically significant symptoms of anxiety, depression and risk of PTSD, and the lowest ratings in the environment domain, compared to physical, psychological and social relations domains (Leiler et al., 2019). The authors suggested that limited healthcare, high levels of passivity and low levels of meaningful daily activities contributed to their poor mental health and perceived quality of life.

Variations and differences in place-specific social determinants of refugee mental health are mediated by networks of transnational, national, governmental and non-governmental organisations. Comparative and longitudinal studies have demonstrated these dynamics and outcomes particularly well. One study compared Syrian refugees in Turkey and Sweden, with the former receiving minimal support and living in abject conditions, and the latter in well-resourced community accommodation with access to language centres, shops, library and leisure facilities, government support and benefits (Cheung Chung et al., 2018). The location of residence significantly correlated with mental health outcomes and co-varied with trauma exposure characteristics and depression. Refugees in Turkey reported significantly higher post-traumatic stress symptoms and psychiatric co-morbidity. When compared to the study of Swedish migration agency's housing facilities (Leiler et al., 2019), these results reinforce the key role of institutional actors in shaping expression of localised social determinants where refugees reside.

A longitudinal study of community-based advocacy and learning interventions, to improve social determinants of refugee mental health in a large Southwestern US city, showed significantly decreased psychological distress and increased quality of life outcomes (Goodkind et al., 2014). In Northern Greece, Beza et al. (2021) found that longer stays for urban refugees combined with a perception that NGOs addressed their needs, meant refugees in urban apartments had better outcomes than camp refugees. Cooperation between UNHCR, the Greek government and local NGOs included legal and psychological support, integration courses, employability and schooling support. Consequently, refugees in urban apartments were exposed to qualitatively better social environment than camp refugees. Studies show that community-based and psychosocial care interventions can create positive mental health outcomes through coordinated and evidence-based work of voluntary and healthcare sectors, educational and artistic institutions and close cooperation between refugees, volunteers, and specialists (Elswick et al., 2021; Zehetmair et al., 2021).

Employment is a particularly salient social determinant of refugee mental health as it is indicative of other social determinants and place-specific factors, such as legal status, access to education and language skills, recognition of pre-flight professional qualifications, and discrimination. According to Blight et al. (2006), higher unemployment in urban areas compared to rural areas exacerbated poor mental health outcomes for refugee men and women who had previously worked for longer periods. Another study comparing refugees in the UK and US, found that refugees in London experienced more issues with legal status, family separation and unemployment (Warfa et al., 2012). Employment had the most impact on reducing the odds of major depression. Occupational downgrading is common among refugees with previous education and professional qualifications not being recognised. Underemployment, unemployment or employment below skill levels affect other areas of settlement and wellbeing (Fozdar, 2009; Papadopoulos et al., 2004; Warfa et al., 2012). For example, refugees in regional Australia who reported significantly poorer quality of life were also more likely to be employed in jobs below their skill levels compared to refugees in urban areas (Correa-Velez et al., 2011). In remote areas, problems with employment and education are also related to travel distances and limited transportation (Ziersch et al., 2020).

#### 3.4.3 Theme 3: Residential instability, post-migration mobility and mental health

Several studies reviewed the importance of residential instability for refugee mental health. A study by Warfa et al. (2006) revealed it took about five years before refugees found permanent accommodation in London. The main causes of residential instability, in their study, were sub-standard and short-term housing, racism, discrimination, and lack of employment opportunities. Another study, Bhui et al. (2012) found that moving within five years of arrival increased risk of psychiatric disorders. This risk was not related to frequency or distance but to being forced to move or having no choice over moving. Some people moved to similar or more deprived areas, to find employment or to be close to friends and relatives - these social support networks appeared protective against psychiatric disorder and distress. Post-migration mobility and access to stable and safe housing is also associated with changes in symptoms of post-traumatic stress. Participants who experienced a forced move reported more severe symptoms a year later, while those who moved out of choice reported fewer symptoms (Gillespie et al., 2020).

Reviews show that refugee children, adolescents and women are particularly vulnerable to the adverse effects of residential instability. According to Warfa et al. (2006) residential instability disrupts schooling, undermines social cohesion, and creates overall psychological distress in refugee families who feel they have no control over life or their future. Moving also disrupted health and mental health service use for those already poorly engaged with UK health services. In the US and Canada, both forced and voluntary post-migration moves were more likely among young families (Gillespie et al., 2020), with involuntary moves associated with greater exposure to violence and concerns about neighbourhood safety impacting children's ability to socialise and form friendships (see also Montgomery, 2008). These studies show that refugee children and adolescents changed schools because of neighbourhood safety concerns and unstable housing.

Residential instability is also linked to safety concerns within refugee communities and family circles, particularly for women and people with mental health issues. In one UK study, refugees with psychiatric disorders were forced to move more often to avoid stigma and hostility (Bhui et al., 2012). Murray et al. (2019) looked at post-migration mobility among refugee women who experienced family and gender-based violence in Australia. The authors compared movement of women between rural and urban areas and the role of localised specialised services for their wellbeing. Proximity to metropolitan centres increased the availability of culturally informed services, but high demand reduced accessibility. Relocating to remote support centres offered longer shelter periods and an escape from community stigma, but increased exposure to local discrimination and reduced access to family support networks.

#### 3.4.4 Theme 4: Ethnic density and diversity and localised support networks

Several studies considered the importance of ethnic diversity for refugee mental health and wellbeing. Rural areas with small residential populations typically have low ethnic diversity, which can be challenging. Refugees resettled in remote rural areas may value a certain level of welfare, job opportunity and economic safety, but feel misunderstood by the locals, including health professionals (Glorius et al., 2020; Grønseth, 2001). For instance, Grønseth (2001) reported difficulties accessing mental health support among Tamil refugees in remote Norwegian fishing villages. Despite an established local refugee community, management of their mental health complaints revolved around the notion of 'cultural differences'. Lack of ethnic diversity can result in social isolation, but this is not confined to rural areas. A study of Bosnian refugees in Chicago, US, showed many felt socially isolated despite a co-ethnic community in the area. Loss of family members and close friends in combination with memories of pre-war life added to the sense of loneliness (Miller et al., 2002).

However, remote, relatively isolated areas can offer some benefits for mental wellbeing. Glorius et al. (2020) suggested that low ethnic diversity can help foster connections with the local people and, if these are positive, improve mental health outcomes, sense of belonging and general wellbeing. Over time, weak social ties between refugees and the locals grew into stronger ties fostered by social proximity, frequent contact, and mutual help. Furthermore, some refugees may seek to distance themselves geographically or socially and prefer relative solitude to engage in personal healing or to avoid prevalent narratives of past trauma (Biglin, 2020; Miller et al., 2002). El-Bialy and Mulay (2015) show how refugees who stayed in a small urban centre in a sparsely populated, remote Canadian province, initially felt isolation, exclusion, and loneliness because of low ethnic diversity in the area. However, some came to value their relative isolation from people of their ethnic because it enabled introspection and personal healing away from dominant communal trauma narratives.

Only one study explicitly used the concept of ethnic density to examine the relation between refugee mental health and place. Finnvold & Ugreninov (2018, p, 45) used the phrase ‘ethnic enclave’ to define an ethnically dense area as a “geographic entity where the share of immigrants with a common country of birth is at least twice as large as that of the same group in the total population, in the same geographic entity”. They compared admission rates to mental health institutions between refugees living inside or outside ethnic enclaves, to areas with or without high ethnic density. The results showed that refugees living in ethnic enclaves had lower admission rates despite higher rates of deprivation characteristic of such ethnic enclaves. According to the authors, ethnic density may work as a buffer against distressing experiences. However, they also questioned if ethnic enclaves might suppress mental health help-seeking among refugees because of intra-community stigma (see also Murray et al., 2019).

#### 3.4.5 Theme 5: Neighbourhood violence and disorder are noted as significant place-related factors in relatively recent literature on refugee mental health

One emerging theme in recent studies is the association between neighbourhood violence and refugee mental health, highlighting exposure to physical and verbal violence, and threats to safety and personal integrity. To account for the effects of neighbourhood violence on refugee mental health, some studies used the concepts of post-resettlement or post-migration trauma (Beiser and Hou, 2016; Kim, 2016; Lincoln et al., 2021). Others describe adverse experiences, including discrimination, and witnessing and experiences of verbal and physical violence (Bhui et al., 2012; Gillespie et al., 2020; Montgomery, 2008; Salhi et al., 2021).

The studies focusing on post-migration trauma suggest this should be understood in cumulative terms together with traumatic experiences of pre-flight and passage (Beiser and Hou, 2016; Miller et al., 2002). According to Beiser and Hou (2016), the key element of post-migration trauma included experiences of discrimination, unfair treatment, verbal and physical attacks. For refugee youth compared to immigrant youth, the combination of pre-flight experience, internment in refugee camps, refugee family dynamics, poverty and higher rates of parental depression contributed to higher self-reported rates of emotional problems and aggressive behaviour. Kim (2016) defined post-resettlement traumas in psychosocial terms, such as seeing people injured or killed and being threatened with a weapon.

Indeed, refugee experiences of settlement and their sense of safety and belonging are associated with location and carry strong implications for mental health and wellbeing. According to Ziersch et al. (2017), place-related factors such as neighbourhood safety and social connections influenced whether refugee housing was suitable or not. High levels of criminal activity and social disorder had adverse effects on mental health. Whereas social connections with 'good neighbours' increased the sense of belonging to the wider community, having positive effects on wellbeing. Other studies showed refugees moved because of neighbourhood violence and discrimination and subsequently reported more severe symptoms of post-traumatic stress (Gillespie et al., 2020; Lincoln et al., 2021; see also Bhui et al., 2012). Exposure to violence predicted poorer mental health outcomes in refugee school children (Montgomery, 2008).

Interestingly, Kearns et al. (2017) found that asylum seekers and refugees who had lived in a deprived area for more than a year had better self-rated health and mental wellbeing than recent arrivals, locals and economic migrants. The authors hypothesised that, on the one hand, refugee settlement with little to no-choice over neighbourhood or area yet with minimal government housing standards, could be psychologically better than a limited choice of substandard living conditions faced by migrants and locals. On the other hand, over time, refugees benefit from settling in areas with access to their cultural communities.

## 4 Discussion

For refugees in the Global North, structural and everyday conditions of post-migration are imbricated with experiences of trauma and flight, and present considerable challenges to mental health and wellbeing. These challenges intertwine with places of refugee residence and their social and material environments. Refugees may have little choice where they resettle and live. Consequently, stressors encountered, such as adverse neighbourhood safety, housing, climate, employment options, living conditions, healthcare, and community ties, have considerable implications for mental health and wellbeing that are situated.

Our scoping review found that very few studies conceptualised place explicitly. Rather, place was broadly conceived to span geographical and spatial dimensions from the micro- (e.g., housing, school and urban allotments) to the macro-level (e.g., countries and cities). Further, studies identified a range of place characteristics considered specifically relevant to refugee mental health and wellbeing (e.g., neighbourhood safety, housing quality and social environment) which suggested that place can be seen as a mediating factor between refugee status, everyday experiences, and mental health. The diversity of people's experiences is beyond our review as our, more modest aim, has been simply to focus on how links between place and refugee mental health have been approached in studies to date (see Table 6). Specifically, our review identified five key themes related to the nexus between place and mental health.

Firstly, several studies paid explicit attention to how physical and material elements of places affected refugees’ mental health and wellbeing. They showed how architecture, urban planning, the natural environment and climate can play a crucial role in mental health, healing and recovery. For instance, refugees can experience rural locations as disorienting and lonely and find harsh climate and weather to be stressful. However, these negative experiences can be offset by social connections with other refugees, local residents and organisations (Herslund and Paulgaard, 2021). In most instances, material and social aspects of place are inter-related but the role of physicality of place is of key importance. There is an emerging work on materiality, embodiment and socio-cultural context that explores the therapeutic potential and pitfalls of physical spaces (Biglin, 2020; 2021). The concept of therapeutic landscapes (Gesler, 1992) used by few studies might prove particularly generative to explore these factors further, showing how therapeutic possibilities of place are not given or self-evident but can emerge over time and in unexpected ways.

Secondly, our review further highlighted that places of refugee residence are governed by localised forms of re-settlement policies and assistance, creating a complex grid of social and economic determinants of mental health specific to locales of residence. Employment, healthcare, welfare, accommodation, and education are the most salient localised determinants of mental health identified by the studies. These factors have cumulative consequences for refugee wellbeing and mental health. For instance, employment was one of the key social place-specific determinants of mental health. In some cases, rural regions with varied industries offer more stable employment opportunities (Blight et al., 2006). However, occupational downgrading is common, and psychosocial and specialised health services are limited in remote areas (Correa-Velez et al., 2011).

Thirdly, studies explored how refugees may move voluntarily to find permanent and safe housing and employment and to seek out cultural resources and co-ethnic community support; and involuntarily to escape areas that present risks of violence, destitution, discrimination and marginalisation, and family and gender-based violence within communities (Murray et al., 2019). Opportunities to move, either voluntarily or involuntarily, appear to be closely related to different social, cultural and economic resources for refugees depending on their gender, age, socio-economic status and education. Residential instability and forced post-migration mobility has been associated with increased risk of mental health problems (Bhui et al., 2012), and frequent moves affect stable access to healthcare services and schooling (Warfa et al., 2006).

Fourthly, our review highlights studies showing that refugees seek to settle close to community networks, indicating the importance of ethnic density and diversity for refugee mental health. Findings show great diversity in patterns of settlement but areas with high ethnic diversity and access to co-ethnic populations are usually reported in inner-city neighbourhoods. While these places offer community support and access to specialised psychosocial services, they also tend to increase exposure to urban violence (Salhi et al., 2021). In contrast, refugee resettlement in rural areas can reduce access to community support and increases risk of discrimination based on refugee identity, but it can offer respite from intra-community stigma and violence (Murray et al., 2019).

Finally, our review suggests that neighbourhood violence and disorder is an emerging area of interest in refugee mental health research along with broader focus on the role of hostility in migrant and ethnic minority mental health (Gillespie et al., 2020; Morgan et al., 2019). Reviewed studies indicate that neighbourhood violence included serious incidents and exposure to discrimination. A related focus has been on neighbourhood disorder, which refers to poor quality of material environment, public disorder, and area deprivation (Ziersch et al., 2017).

Focusing on place brings different layers of refugee settlement and everyday lives together, allowing a more holistic understanding of the situated and dynamic mental health experiences of refugees in Global North countries. Place should be seen as multifactorial construct that embeds the post-migration context of refugee mental health within a broader asylum landscape. Advances in this area could be made by more detailed accounts of the role of place in reported findings. Building on these insights, it is important to conduct more research on both negative and sought-after aspects of relative isolation, social or geographical or both. Relatedly, more detailed attention should be paid to how places change over time and how it intersects with longer term refugee mental health outcomes. Noticeably little attention has been paid to how cultural variation or cultural background might mediate relations between place and refugee mental health. This is surprising given the connection of mental health and culture and the nature of psychiatric conditions (Kirmayer and Gómez-Carrillo, 2019). Culture is an inherent factor for refugee mental health and place shaping all aspects of post-migration context, from community support to social integration and discrimination.

Our findings are relevant for dispersal policies implemented in many Global North countries whereby people seeking asylum receive conditional accommodation and financial support outside large urban centres. For instance, in Scandinavian countries, they are meant to reduce the formation of ethnic enclaves in larger cities and foster social integration into the host society, whereas in the UK, they are intended to relieve London and South East England from housing and social-economic pressures (Larsen, 2011; Stewart, 2012). Although claimed to have these aims, dispersal policies can actually reproduce or exacerbate inequalities between refugees and the rest of the population (Valenta and Bunar, 2010; Zetter et al., 2005). Refugees may find themselves living in deprived or remote areas with limited options to seek employment, build social capital and develop a sense of belonging (Darling, 2016, 2022; Phillimore and Goodson, 2006), Therefore, to address the underlying causes of refugee mental health problems might need social rather than medical solutions in the first instance. That is, rather than starting with the provision of specialised mental health interventions, it might be advisable to provide community support and opportunities to develop diaspora connection for positive social integration first (Campbell and Burgess, 2012; Silove et al., 2017; Soltan et al., 2020). Such community-based mental health support emphasises the importance of tackling social determinants of health, i.e., the conditions where people are born, live, learn, work, play, worship and age that affect their health, including their mental health (Campbell et al., 2018; Ellis et al., 2010; Kanagaratnam et al., 2020; Kienzler, 2008; Marmot, 2005).

Supporting refugees through community-based projects helps address psychological trauma within the context of post-migration difficulties and fosters meaningful social integration (de Smet et al., 2019; Elswick et al., 2021). This is particularly pertinent given that migrants in general can transform local areas and economies, creating better and more prosperous living environments for all (Hall, 2012; Phillimore and Goodson, 2006). The ethnic density effect, associated with better mental health outcomes and reduced risk of psychosis, is well established in migrant and minority ethnic populations in neighbourhood settings (Schofield et al., 2017). However, it is relatively under-researched in refugees. Likewise, better understanding of neighbourhood safety and disorder (Polling et al., 2014) in conjunction with characteristics of place such as ethnic density and diversity, could offer valuable insights into the dynamics and complexities of protective and harmful effects for refugee mental health. Future research should focus on how these factors and patterns are distributed across different places and how they change over time; how and why refugees move between different places, and what the implications are for their mental health.

## 5 Limitations

There are several limitations to this scoping review. Firstly, we only focused on Global North and high-income countries. Including all countries would provide a broader assessment of available evidence and, potentially, expand the range of place-specific factors researched in low- and middle-income contexts. We also excluded refugee camps as specific locations conceptualised as cities in some studies (Agier, 2002; Sanyal, 2012) as well as asylum seekers, whose status and circumstances are qualitatively different. Secondly, we included studies that consider place indirectly, potentially missing relevant articles that could be included according to this relatively arbitrary criterion. Thirdly, inclusion of studies published only in English leaves out research reported in other languages and, therefore, may miss reporting other place-related factors and themes. Finally, our focus was not on quality assessment of the studies and available evidence, but on the exploration and description of how place and its characteristics are discussed in the literature.

## 6 Conclusion

In this scoping review we present 34 studies on the role of place in refugee mental health. These follow a range of methodological approaches and varying conceptions of both mental health and place. While this is still very much a nascent field of enquiry, we found several consistent themes centring on: the material and physical aspects of place; place-specific social determinants, such as employment opportunities and institutional support; experience of residential instability and mobility; the importance of ethnic density and localised social support networks; and lastly, recent work on neighbourhood violence and disorder. These provide a useful starting point for future research in this developing field.

## Funding

This work was supported by the 10.13039/501100000265UK Medical Research Council [MR/S025510/1].

## Declaration of competing interest

None.

## Footnotes

1 While we recognise the complexity of this terminology, by Global North we refer to high-income regions such as Europe, North America and Australia. For the list of countries included in this review, see the Results section.
